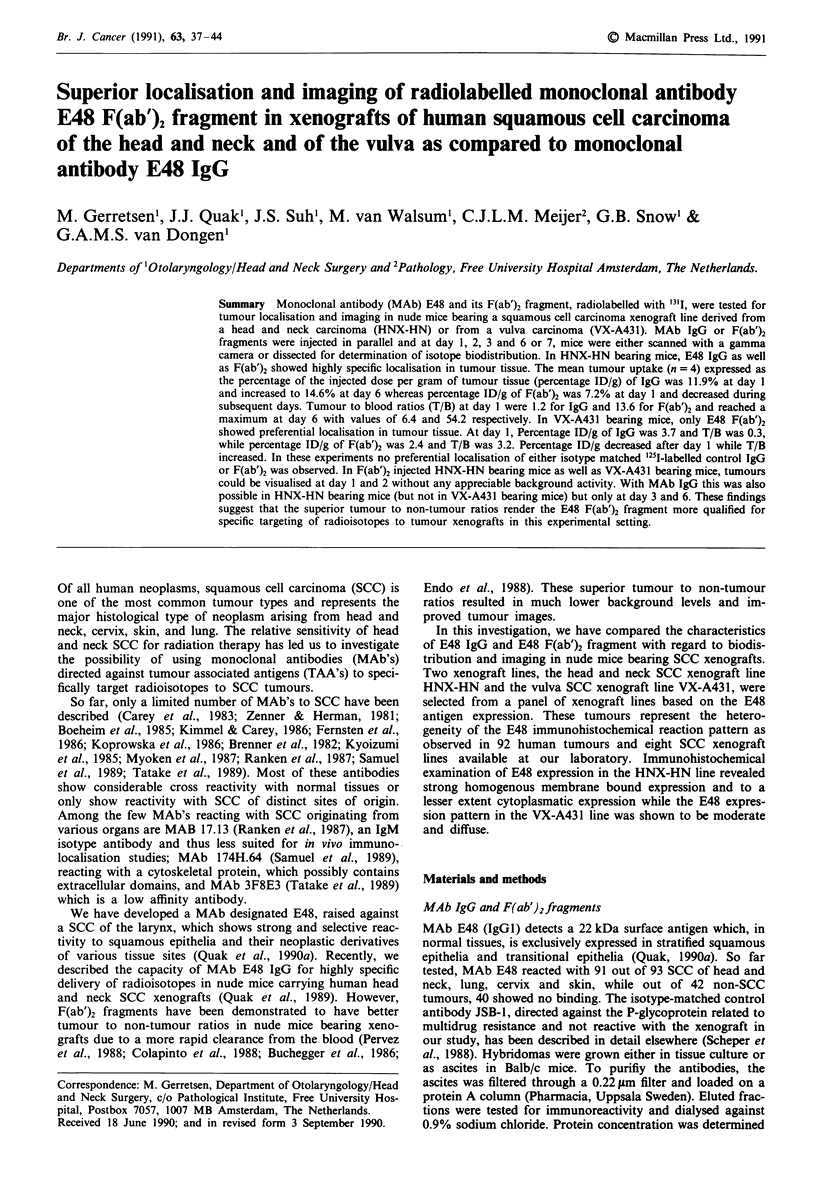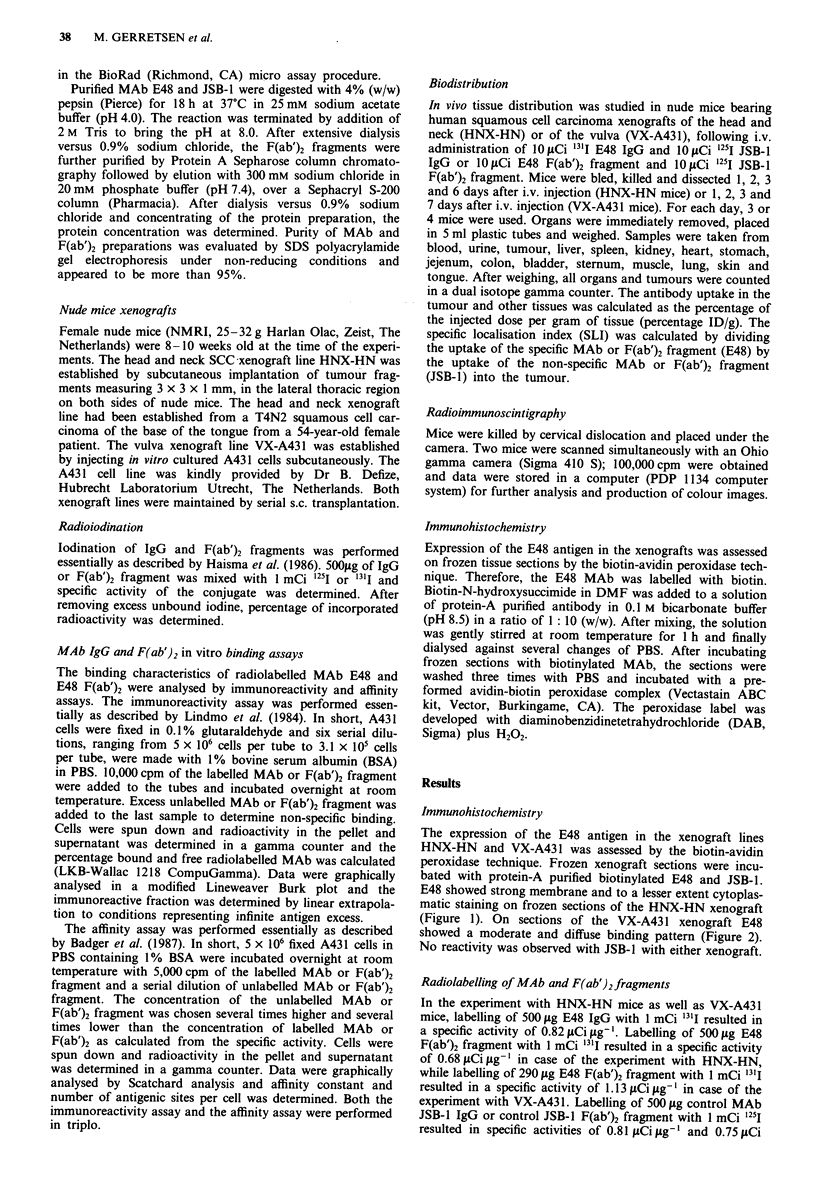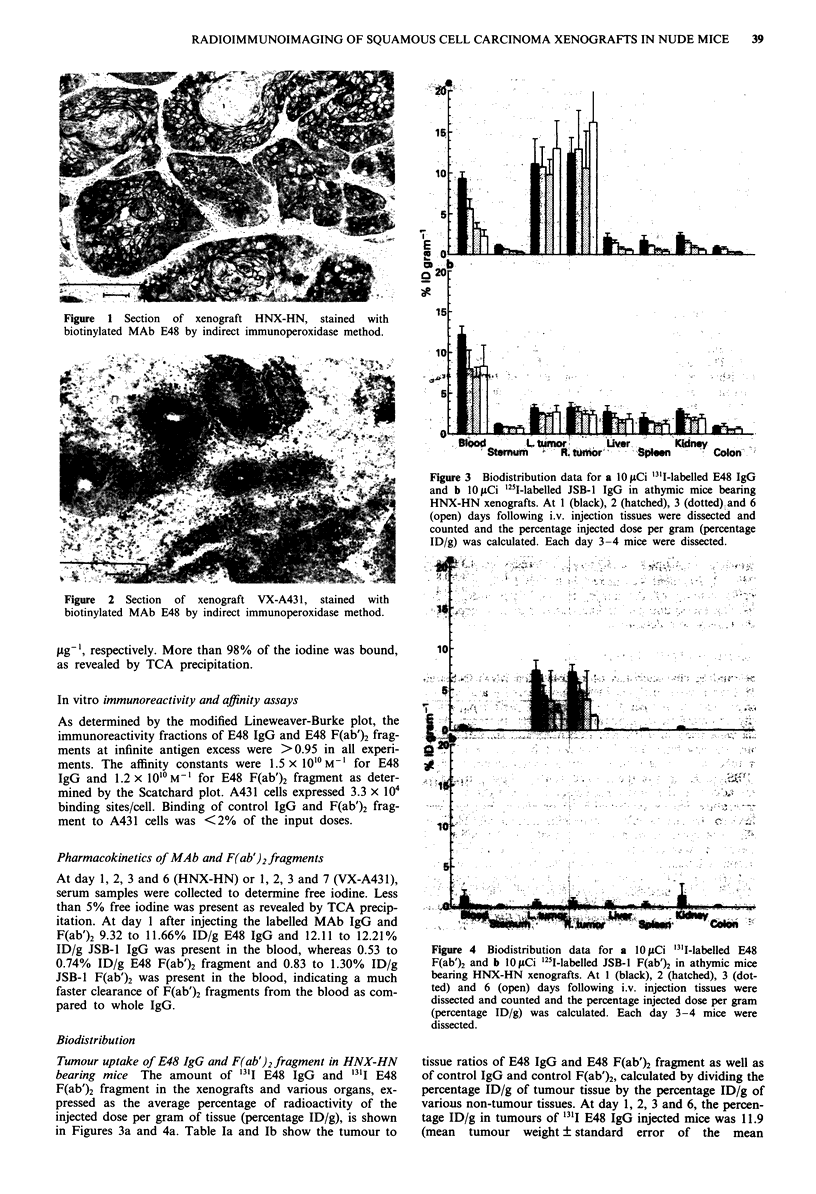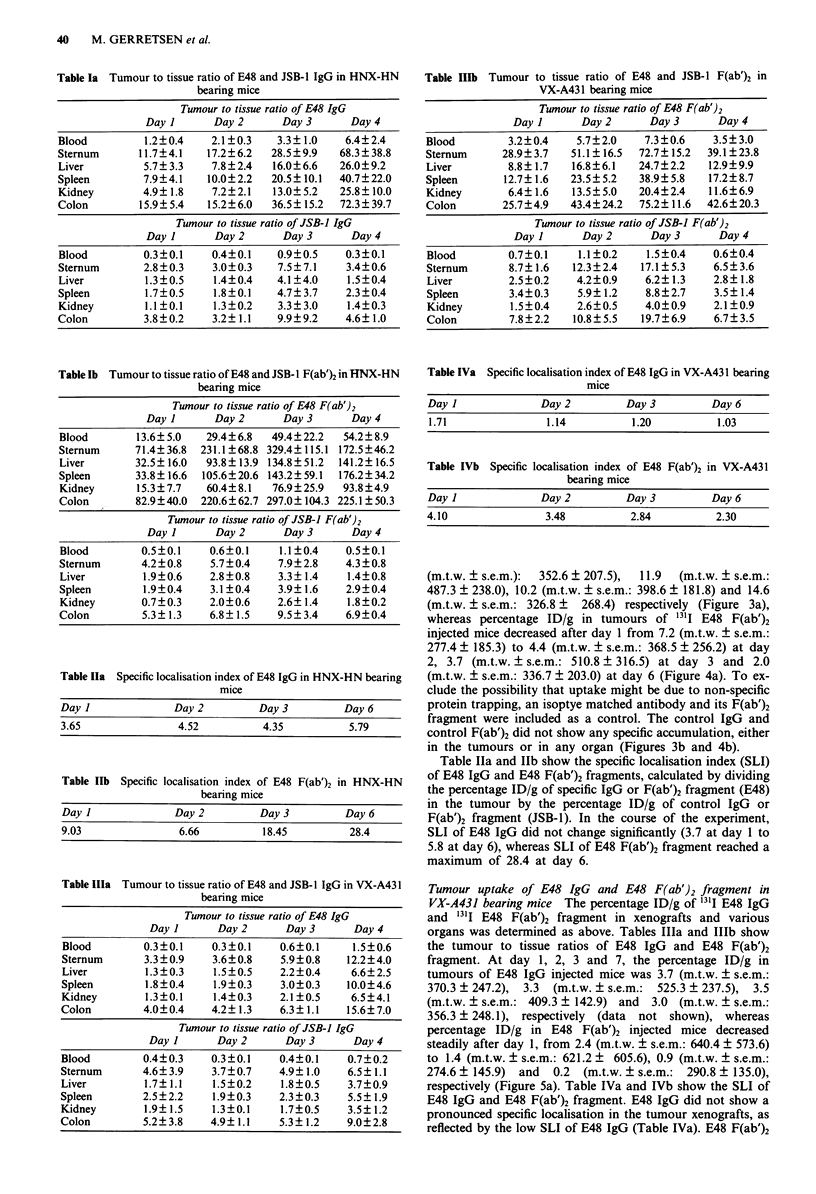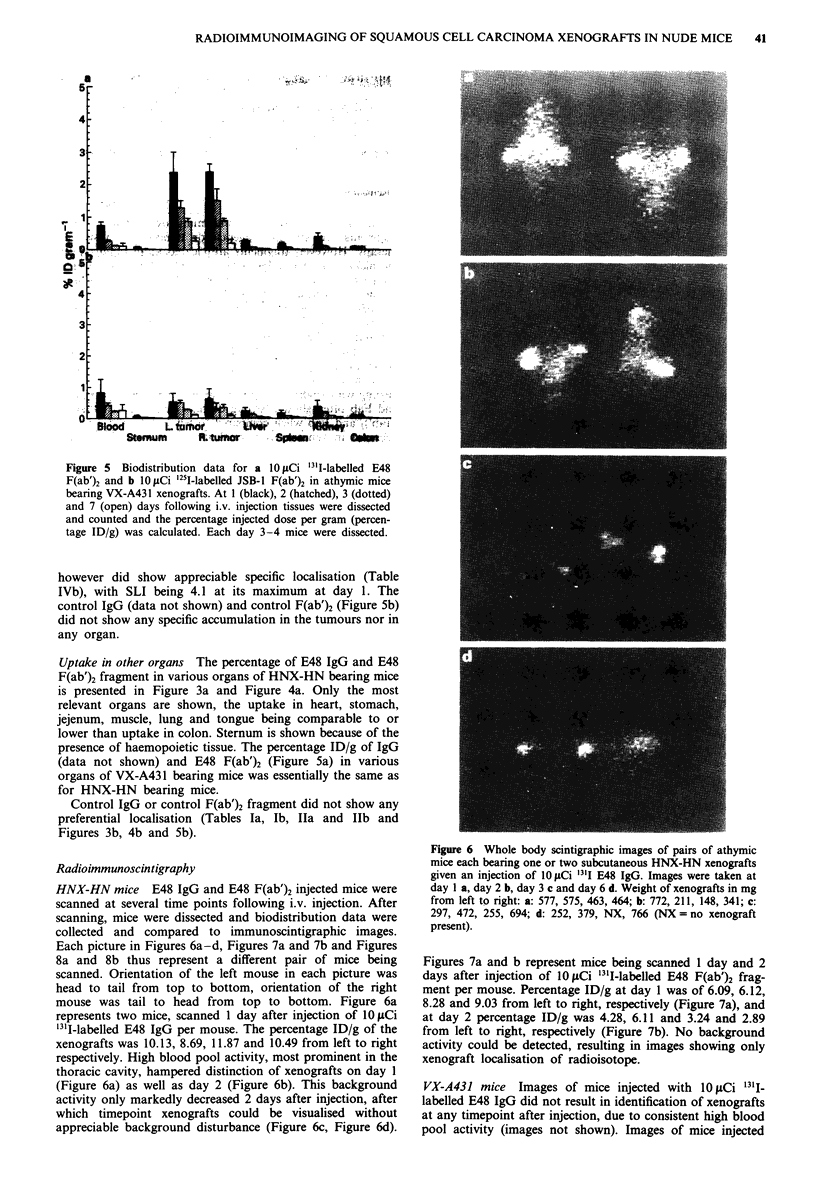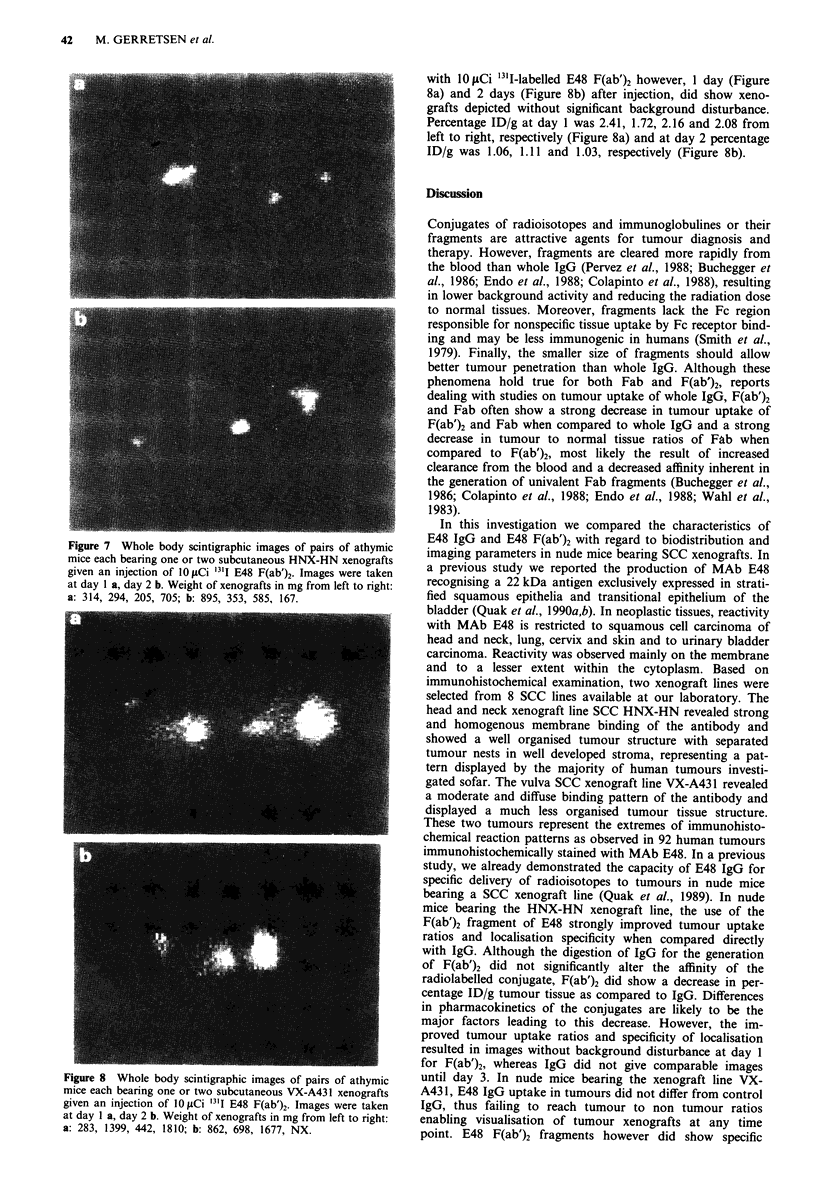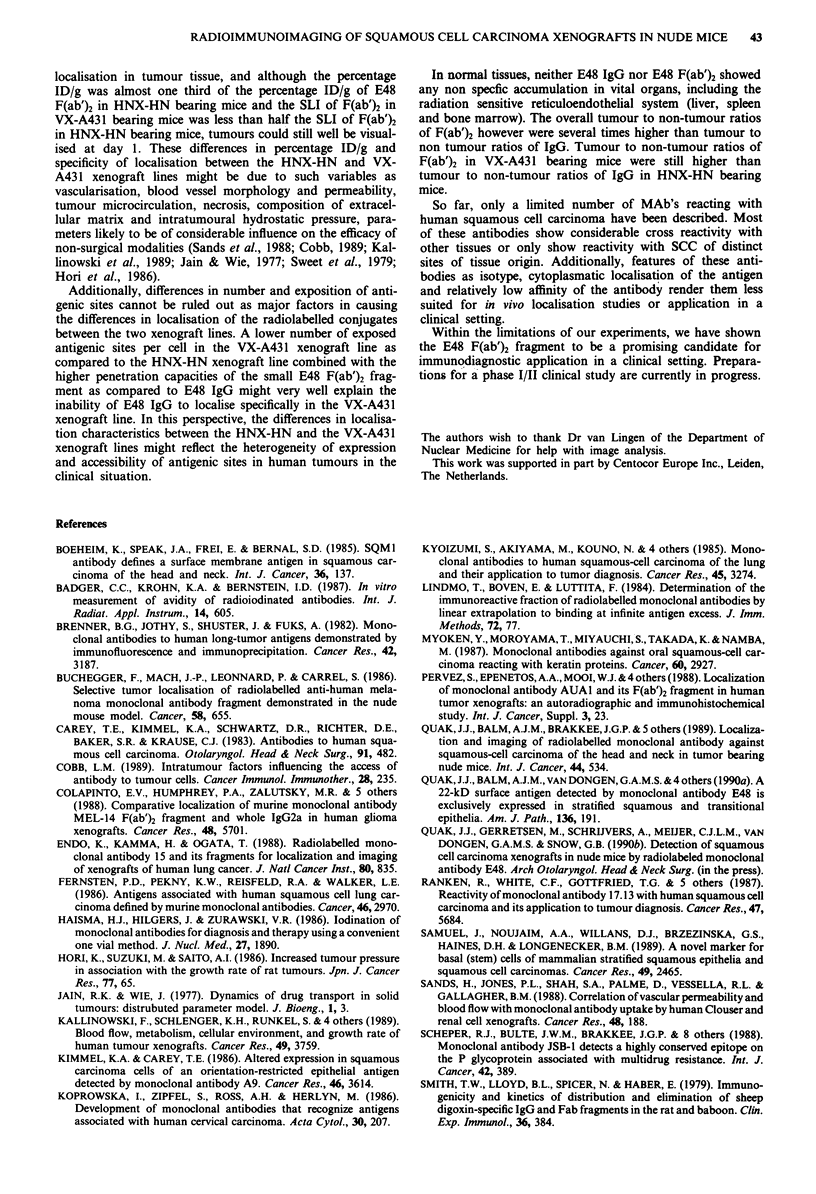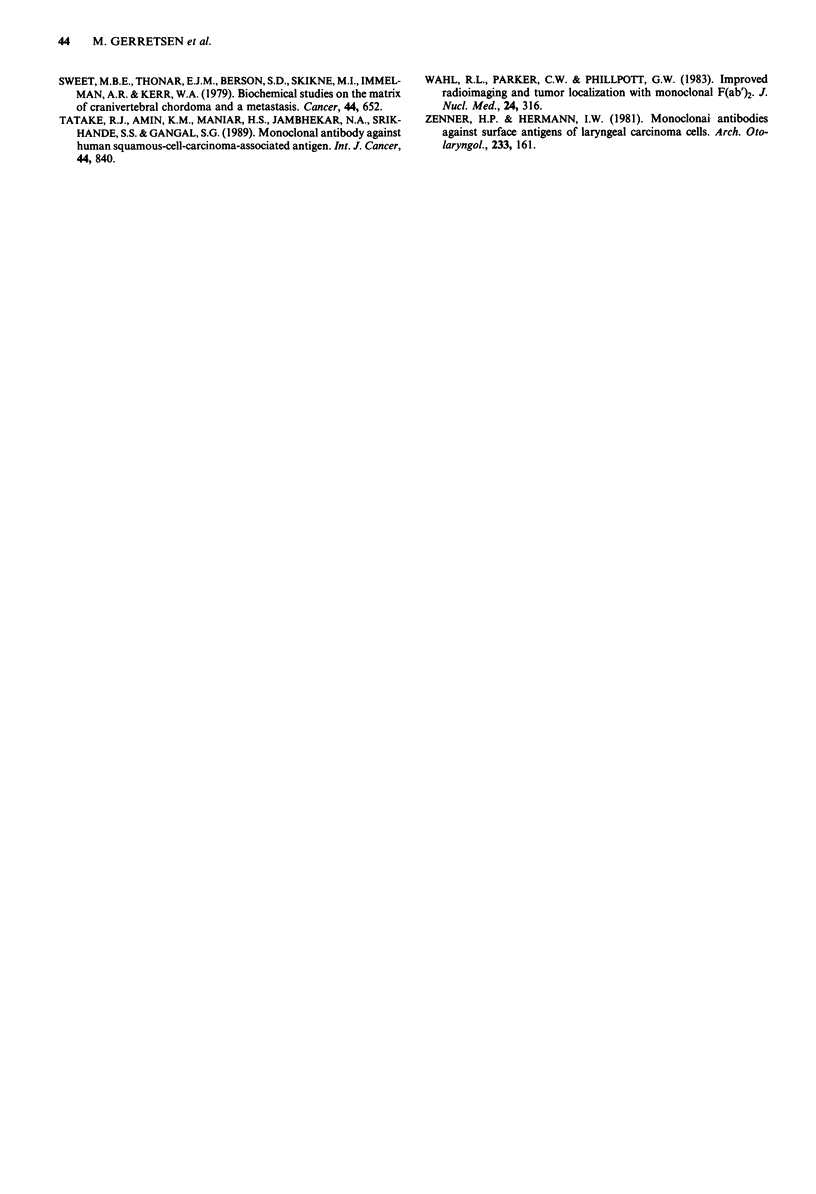# Superior localisation and imaging of radiolabelled monoclonal antibody E48 F(ab')2 fragment in xenografts of human squamous cell carcinoma of the head and neck and of the vulva as compared to monoclonal antibody E48 IgG.

**DOI:** 10.1038/bjc.1991.9

**Published:** 1991-01

**Authors:** M. Gerretsen, J. J. Quak, J. S. Suh, M. van Walsum, C. J. Meijer, G. B. Snow, G. A. van Dongen

**Affiliations:** Department of Otolaryngology/Head and Neck Surgery, Free University Hospital Amsterdam, The Netherlands.

## Abstract

**Images:**


Br. J. Cancer (1991), 63, 37-44 © Macmillan Press Ltd., 1991

### Superior localisation and imaging of radiolabelled monoclonal antibody E48 F(ab')$_2$ fragment in xenografts of human squamous cell carcinoma of the head and neck and of the vulva as compared to monoclonal antibody E48 IgG

M. Gerretsen[1], J.J. Quak[1], J.S. Suh[1], M. van Walsum[1], C.J.L.M. Meijer[2], G.B. Snow[1] & G.A.M.S. van Dongen[1]

*Departments of [1]Otolaryngology/Head and Neck Surgery and [2]Pathology, Free University Hospital Amsterdam, The Netherlands.*

**Summary** Monoclonal antibody (MAb) E48 and its F(ab')$_2$ fragment, radiolabelled with $^{131}$I, were tested for tumour localisation and imaging in nude mice bearing a squamous cell carcinoma xenograft line derived from a head and neck carcinoma (HNX-HN) or from a vulva carcinoma (VX-A431). MAb IgG or F(ab')$_2$ fragments were injected in parallel and at day 1, 2, 3 and 6 or 7, mice were either scanned with a gamma camera or dissected for determination of isotope biodistribution. In HNX-HN bearing mice, E48 IgG as well as F(ab')$_2$ showed highly specific localisation in tumour tissue. The mean tumour uptake ($n = 4$) expressed as the percentage of the injected dose per gram of tumour tissue (percentage ID/g) of IgG was 11.9% at day 1 and increased to 14.6% at day 6 whereas percentage ID/g of F(ab')$_2$ was 7.2% at day 1 and decreased during subsequent days. Tumour to blood ratios (T/B) at day 1 were 1.2 for IgG and 13.6 for F(ab')$_2$ and reached a maximum at day 6 with values of 6.4 and 54.2 respectively. In VX-A431 bearing mice, only E48 F(ab')$_2$ showed preferential localisation in tumour tissue. At day 1, Percentage ID/g of IgG was 3.7 and T/B was 0.3, while percentage ID/g of F(ab')$_2$ was 2.4 and T/B was 3.2. Percentage ID/g decreased after day 1 while T/B increased. In these experiments no preferential localisation of either isotype matched $^{125}$I-labelled control IgG or F(ab')$_2$ was observed. In F(ab')$_2$ injected HNX-HN bearing mice as well as VX-A431 bearing mice, tumours could be visualised at day 1 and 2 without any appreciable background activity. With MAb IgG this was also possible in HNX-HN bearing mice (but not in VX-A431 bearing mice) but only at day 3 and 6. These findings suggest that the superior tumour to non-tumour ratios render the E48 F(ab')$_2$ fragment more qualified for specific targeting of radioisotopes to tumour xenografts in this experimental setting.

Of all human neoplasms, squamous cell carcinoma (SCC) is one of the most common tumour types and represents the major histological type of neoplasm arising from head and neck, cervix, skin, and lung. The relative sensitivity of head and neck SCC for radiation therapy has led us to investigate the possibility of using monoclonal antibodies (MAb's) directed against tumour associated antigens (TAA's) to specifically target radioisotopes to SCC tumours.

So far, only a limited number of MAb's to SCC have been described (Carey *et al.*, 1983; Zenner & Herman, 1981; Boeheim *et al.*, 1985; Kimmel & Carey, 1986; Fernsten *et al.*, 1986; Koprowska *et al.*, 1986; Brenner *et al.*, 1982; Kyoizumi *et al.*, 1985; Myoken *et al.*, 1987; Ranken *et al.*, 1987; Samuel *et al.*, 1989; Tatake *et al.*, 1989). Most of these antibodies show considerable cross reactivity with normal tissues or only show reactivity with SCC of distinct sites of origin. Among the few MAb's reacting with SCC originating from various organs are MAB 17.13 (Ranken *et al.*, 1987), an IgM isotype antibody and thus less suited for *in vivo* immunolocalisation studies; MAb 174H.64 (Samuel *et al.*, 1989), reacting with a cytoskeletal protein, which possibly contains extracellular domains, and MAb 3F8E3 (Tatake *et al.*, 1989) which is a low affinity antibody.

We have developed a MAb designated E48, raised against a SCC of the larynx, which shows strong and selective reactivity to squamous epithelia and their neoplastic derivatives of various tissue sites (Quak *et al.*, 1990*a*). Recently, we described the capacity of MAb E48 IgG for highly specific delivery of radioisotopes in nude mice carrying human head and neck SCC xenografts (Quak *et al.*, 1989). However, F(ab')$_2$ fragments have been demonstrated to have better tumour to non-tumour ratios in nude mice bearing xenografts due to a more rapid clearance from the blood (Pervez *et al.*, 1988; Colapinto *et al.*, 1988; Buchegger *et al.*, 1986; Endo *et al.*, 1988). These superior tumour to non-tumour ratios resulted in much lower background levels and improved tumour images.

In this investigation, we have compared the characteristics of E48 IgG and E48 F(ab')$_2$ fragment with regard to biodistribution and imaging in nude mice bearing SCC xenografts. Two xenograft lines, the head and neck SCC xenograft line HNX-HN and the vulva SCC xenograft line VX-A431, were selected from a panel of xenograft lines based on the E48 antigen expression. These tumours represent the heterogeneity of the E48 immunohistochemical reaction pattern as observed in 92 human tumours and eight SCC xenograft lines available at our laboratory. Immunohistochemical examination of E48 expression in the HNX-HN line revealed strong homogenous membrane bound expression and to a lesser extent cytoplasmatic expression while the E48 expression pattern in the VX-A431 line was shown to be moderate and diffuse.

Correspondence: M. Gerretsen, Department of Otolaryngology/Head and Neck Surgery, c/o Pathological Institute, Free University Hospital, Postbox 7057, 1007 MB Amsterdam, The Netherlands.
Received 18 June 1990; and in revised form 3 September 1990.

### Materials and methods

### *MAb IgG and F(ab')$_2$ fragments*

MAb E48 (IgG1) detects a 22 kDa surface antigen which, in normal tissues, is exclusively expressed in stratified squamous epithelia and transitional epithelia (Quak, 1990*a*). So far tested, MAb E48 reacted with 91 out of 93 SCC of head and neck, lung, cervix and skin, while out of 42 non-SCC tumours, 40 showed no binding. The isotype-matched control antibody JSB-1, directed against the P-glycoprotein related to multidrug resistance and not reactive with the xenograft in our study, has been described in detail elsewhere (Scheper *et al.*, 1988). Hybridomas were grown either in tissue culture or as ascites in Balb/c mice. To purifiy the antibodies, the ascites was filtered through a 0.22 µm filter and loaded on a protein A column (Pharmacia, Uppsala Sweden). Eluted fractions were tested for immunoreactivity and dialysed against 0.9% sodium chloride. Protein concentration was determined in the BioRad (Richmond, CA) micro assay procedure.

Purified MAb E48 and JSB-1 were digested with 4% (w/w) pepsin (Pierce) for 18 h at 37°C in 25 mM sodium acetate buffer (pH 4.0). The reaction was terminated by addition of 2 M Tris to bring the pH at 8.0. After extensive dialysis versus 0.9% sodium chloride, the $F(ab')_2$ fragments were further purified by Protein A Sepharose column chromatography followed by elution with 300 mM sodium chloride in 20 mM phosphate buffer (pH 7.4), over a Sephacryl S-200 column (Pharmacia). After dialysis versus 0.9% sodium chloride and concentrating of the protein preparation, the protein concentration was determined. Purity of MAb and $F(ab')_2$ preparations was evaluated by SDS polyacrylamide gel electrophoresis under non-reducing conditions and appeared to be more than 95%.

*Nude mice xenografts*

Female nude mice (NMRI, 25–32 g Harlan Olac, Zeist, The Netherlands) were 8–10 weeks old at the time of the experiments. The head and neck SCC xenograft line HNX-HN was established by subcutaneous implantation of tumour fragments measuring 3 × 3 × 1 mm, in the lateral thoracic region on both sides of nude mice. The head and neck xenograft line had been established from a T4N2 squamous cell carcinoma of the base of the tongue from a 54-year-old female patient. The vulva xenograft line VX-A431 was established by injecting *in vitro* cultured A431 cells subcutaneously. The A431 cell line was kindly provided by Dr B. Defize, Hubrecht Laboratorium Utrecht, The Netherlands. Both xenograft lines were maintained by serial s.c. transplantation.

*Radioiodination*

Iodination of IgG and $F(ab')_2$ fragments was performed essentially as described by Haisma *et al.* (1986). 500μg of IgG or $F(ab')_2$ fragment was mixed with 1 mCi $^{125}I$ or $^{131}I$ and specific activity of the conjugate was determined. After removing excess unbound iodine, percentage of incorporated radioactivity was determined.

*MAb IgG and $F(ab')_2$ in* vitro *binding assays*

The binding characteristics of radiolabelled MAb E48 and E48 $F(ab')_2$ were analysed by immunoreactivity and affinity assays. The immunoreactivity assay was performed essentially as described by Lindmo *et al.* (1984). In short, A431 cells were fixed in 0.1% glutaraldehyde and six serial dilutions, ranging from $5 \times 10^6$ cells per tube to $3.1 \times 10^5$ cells per tube, were made with 1% bovine serum albumin (BSA) in PBS. 10,000 cpm of the labelled MAb or $F(ab')_2$ fragment were added to the tubes and incubated overnight at room temperature. Excess unlabelled MAb or $F(ab')_2$ fragment was added to the last sample to determine non-specific binding. Cells were spun down and radioactivity in the pellet and supernatant was determined in a gamma counter and the percentage bound and free radiolabelled MAb was calculated (LKB-Wallac 1218 CompuGamma). Data were graphically analysed in a modified Lineweaver Burk plot and the immunoreactive fraction was determined by linear extrapolation to conditions representing infinite antigen excess.

The affinity assay was performed essentially as described by Badger *et al.* (1987). In short, $5 \times 10^6$ fixed A431 cells in PBS containing 1% BSA were incubated overnight at room temperature with 5,000 cpm of the labelled MAb or $F(ab')_2$ fragment and a serial dilution of unlabelled MAb or $F(ab')_2$ fragment. The concentration of the unlabelled MAb or $F(ab')_2$ fragment was chosen several times higher and several times lower than the concentration of labelled MAb or $F(ab')_2$ as calculated from the specific activity. Cells were spun down and radioactivity in the pellet and supernatant was determined in a gamma counter. Data were graphically analysed by Scatchard analysis and affinity constant and number of antigenic sites per cell was determined. Both the immunoreactivity assay and the affinity assay were performed in triplo.

*Biodistribution*

*In vivo* tissue distribution was studied in nude mice bearing human squamous cell carcinoma xenografts of the head and neck (HNX-HN) or of the vulva (VX-A431), following i.v. administration of 10 μCi $^{131}I$ E48 IgG and 10 μCi $^{125}I$ JSB-1 IgG or 10 μCi E48 $F(ab')_2$ fragment and 10 μCi $^{125}I$ JSB-1 $F(ab')_2$ fragment. Mice were bled, killed and dissected 1, 2, 3 and 6 days after i.v. injection (HNX-HN mice) or 1, 2, 3 and 7 days after i.v. injection (VX-A431 mice). For each day, 3 or 4 mice were used. Organs were immediately removed, placed in 5 ml plastic tubes and weighed. Samples were taken from blood, urine, tumour, liver, spleen, kidney, heart, stomach, jejenum, colon, bladder, sternum, muscle, lung, skin and tongue. After weighing, all organs and tumours were counted in a dual isotope gamma counter. The antibody uptake in the tumour and other tissues was calculated as the percentage of the injected dose per gram of tissue (percentage ID/g). The specific localisation index (SLI) was calculated by dividing the uptake of the specific MAb or $F(ab')_2$ fragment (E48) by the uptake of the non-specific MAb or $F(ab')_2$ fragment (JSB-1) into the tumour.

*Radioimmunoscintigraphy*

Mice were killed by cervical dislocation and placed under the camera. Two mice were scanned simultaneously with an Ohio gamma camera (Sigma 410 S); 100,000 cpm were obtained and data were stored in a computer (PDP 1134 computer system) for further analysis and production of colour images.

*Immunohistochemistry*

Expression of the E48 antigen in the xenografts was assessed on frozen tissue sections by the biotin-avidin peroxidase technique. Therefore, the E48 MAb was labelled with biotin. Biotin-N-hydroxysuccimide in DMF was added to a solution of protein-A purified antibody in 0.1 M bicarbonate buffer (pH 8.5) in a ratio of 1 : 10 (w/w). After mixing, the solution was gently stirred at room temperature for 1 h and finally dialysed against several changes of PBS. After incubating frozen sections with biotinylated MAb, the sections were washed three times with PBS and incubated with a preformed avidin-biotin peroxidase complex (Vectastain ABC kit, Vector, Burkingame, CA). The peroxidase label was developed with diaminobenzidinetetrahydrochloride (DAB, Sigma) plus $H_2O_2$.

### Results

*Immunohistochemistry*

The expression of the E48 antigen in the xenograft lines HNX-HN and VX-A431 was assessed by the biotin-avidin peroxidase technique. Frozen xenograft sections were incubated with protein-A purified biotinylated E48 and JSB-1. E48 showed strong membrane and to a lesser extent cytoplasmatic staining on frozen sections of the HNX-HN xenograft (Figure 1). On sections of the VX-A431 xenograft E48 showed a moderate and diffuse binding pattern (Figure 2). No reactivity was observed with JSB-1 with either xenograft.

*Radiolabelling of MAb and $F(ab')_2$ fragments*

In the experiment with HNX-HN mice as well as VX-A431 mice, labelling of 500 μg E48 IgG with 1 mCi $^{131}I$ resulted in a specific activity of 0.82 μCi $\mu g^{-1}$. Labelling of 500 μg E48 $F(ab')_2$ fragment with 1 mCi $^{131}I$ resulted in a specific activity of 0.68 μCi $\mu g^{-1}$ in case of the experiment with HNX-HN, while labelling of 290 μg E48 $F(ab')_2$ fragment with 1 mCi $^{131}I$ resulted in a specific activity of 1.13 μCi $\mu g^{-1}$ in case of the experiment with VX-A431. Labelling of 500 μg control MAb JSB-1 IgG or control JSB-1 $F(ab')_2$ fragment with 1 mCi $^{125}I$ resulted in specific activities of 0.81 μCi $\mu g^{-1}$ and 0.75 μCi

**Figure 1** Section of xenograft HNX-HN, stained with biotinylated MAb E48 by indirect immunoperoxidase method.

**Figure 2** Section of xenograft VX-A431, stained with biotinylated MAb E48 by indirect immunoperoxidase method.

$\mu g^{-1}$, respectively. More than 98% of the iodine was bound, as revealed by TCA precipitation.

### *In vitro immunoreactivity and affinity assays*

As determined by the modified Lineweaver-Burke plot, the immunoreactivity fractions of E48 IgG and E48 $F(ab')_2$ fragments at infinite antigen excess were $>0.95$ in all experiments. The affinity constants were $1.5 \times 10^{10}\ M^{-1}$ for E48 IgG and $1.2 \times 10^{10}\ M^{-1}$ for E48 $F(ab')_2$ fragment as determined by the Scatchard plot. A431 cells expressed $3.3 \times 10^4$ binding sites/cell. Binding of control IgG and $F(ab')_2$ fragment to A431 cells was $<2\%$ of the input doses.

### *Pharmacokinetics of MAb and $F(ab')_2$ fragments*

At day 1, 2, 3 and 6 (HNX-HN) or 1, 2, 3 and 7 (VX-A431), serum samples were collected to determine free iodine. Less than 5% free iodine was present as revealed by TCA precipitation. At day 1 after injecting the labelled MAb IgG and $F(ab')_2$ 9.32 to 11.66% ID/g E48 IgG and 12.11 to 12.21% ID/g JSB-1 IgG was present in the blood, whereas 0.53 to 0.74% ID/g E48 $F(ab')_2$ fragment and 0.83 to 1.30% ID/g JSB-1 $F(ab')_2$ was present in the blood, indicating a much faster clearance of $F(ab')_2$ fragments from the blood as compared to whole IgG.

**Figure 3** Biodistribution data for **a** 10 $\mu$Ci $^{131}$I-labelled E48 IgG and **b** 10 $\mu$Ci $^{125}$I-labelled JSB-1 IgG in athymic mice bearing HNX-HN xenografts. At 1 (black), 2 (hatched), 3 (dotted) and 6 (open) days following i.v. injection tissues were dissected and counted and the percentage injected dose per gram (percentage ID/g) was calculated. Each day 3–4 mice were dissected.

**Figure 4** Biodistribution data for **a** 10 $\mu$Ci $^{131}$I-labelled E48 $F(ab')_2$ and **b** 10 $\mu$Ci $^{125}$I-labelled JSB-1 $F(ab')_2$ in athymic mice bearing HNX-HN xenografts. At 1 (black), 2 (hatched), 3 (dotted) and 6 (open) days following i.v. injection tissues were dissected and counted and the percentage injected dose per gram (percentage ID/g) was calculated. Each day 3–4 mice were dissected.

### *Biodistribution*

*Tumour uptake of E48 IgG and $F(ab')_2$ fragment in HNX-HN bearing mice* The amount of $^{131}$I E48 IgG and $^{131}$I E48 $F(ab')_2$ fragment in the xenografts and various organs, expressed as the average percentage of radioactivity of the injected dose per gram of tissue (percentage ID/g), is shown in Figures 3a and 4a. Table Ia and Ib show the tumour to tissue ratios of E48 IgG and E48 $F(ab')_2$ fragment as well as of control IgG and control $F(ab')_2$, calculated by dividing the percentage ID/g of tumour tissue by the percentage ID/g of various non-tumour tissues. At day 1, 2, 3 and 6, the percentage ID/g in tumours of $^{131}$I E48 IgG injected mice was 11.9 (mean tumour weight $\pm$ standard error of the mean

**Table Ia** Tumour to tissue ratio of E48 and JSB-1 IgG in HNX-HN bearing mice

| | Tumour to tissue ratio of E48 IgG | | | |
|---|---|---|---|---|
| | Day 1 | Day 2 | Day 3 | Day 4 |
| Blood | 1.2±0.4 | 2.1±0.3 | 3.3±1.0 | 6.4±2.4 |
| Sternum | 11.7±4.1 | 17.2±6.2 | 28.5±9.9 | 68.3±38.8 |
| Liver | 5.7±3.3 | 7.8±2.4 | 16.0±6.6 | 26.0±9.2 |
| Spleen | 7.9±4.1 | 10.0±2.2 | 20.5±10.1 | 40.7±22.0 |
| Kidney | 4.9±1.8 | 7.2±2.1 | 13.0±5.2 | 25.8±10.0 |
| Colon | 15.9±5.4 | 15.2±6.0 | 36.5±15.2 | 72.3±39.7 |
| | Tumour to tissue ratio of JSB-1 IgG | | | |
| | Day 1 | Day 2 | Day 3 | Day 4 |
| Blood | 0.3±0.1 | 0.4±0.1 | 0.9±0.5 | 0.3±0.1 |
| Sternum | 2.8±0.3 | 3.0±0.3 | 7.5±7.1 | 3.4±0.6 |
| Liver | 1.3±0.5 | 1.4±0.4 | 4.1±4.0 | 1.5±0.4 |
| Spleen | 1.7±0.5 | 1.8±0.1 | 4.7±3.7 | 2.3±0.4 |
| Kidney | 1.1±0.1 | 1.3±0.2 | 3.3±3.0 | 1.4±0.3 |
| Colon | 3.8±0.2 | 3.2±1.1 | 9.9±9.2 | 4.6±1.0 |

**Table Ib** Tumour to tissue ratio of E48 and JSB-1 $F(ab')_2$ in HNX-HN bearing mice

| | Tumour to tissue ratio of E48 $F(ab')_2$ | | | |
|---|---|---|---|---|
| | Day 1 | Day 2 | Day 3 | Day 4 |
| Blood | 13.6±5.0 | 29.4±6.8 | 49.4±22.2 | 54.2±8.9 |
| Sternum | 71.4±36.8 | 231.1±68.8 | 329.4±115.1 | 172.5±46.2 |
| Liver | 32.5±16.0 | 93.8±13.9 | 134.8±51.2 | 141.2±16.5 |
| Spleen | 33.8±16.6 | 105.6±20.6 | 143.2±59.1 | 176.2±34.2 |
| Kidney | 15.3±7.7 | 60.4±8.1 | 76.9±25.9 | 93.8±4.9 |
| Colon | 82.9±40.0 | 220.6±62.7 | 297.0±104.3 | 225.1±50.3 |
| | Tumour to tissue ratio of JSB-1 $F(ab')_2$ | | | |
| | Day 1 | Day 2 | Day 3 | Day 4 |
| Blood | 0.5±0.1 | 0.6±0.1 | 1.1±0.4 | 0.5±0.1 |
| Sternum | 4.2±0.8 | 5.7±0.4 | 7.9±2.8 | 4.3±0.8 |
| Liver | 1.9±0.6 | 2.8±0.8 | 3.3±1.4 | 1.4±0.8 |
| Spleen | 1.9±0.4 | 3.1±0.4 | 3.9±1.6 | 2.9±0.4 |
| Kidney | 0.7±0.3 | 2.0±0.6 | 2.6±1.4 | 1.8±0.2 |
| Colon | 5.3±1.3 | 6.8±1.5 | 9.5±3.4 | 6.9±0.4 |

**Table IIa** Specific localisation index of E48 IgG in HNX-HN bearing mice

| Day 1 | Day 2 | Day 3 | Day 6 |
|---|---|---|---|
| 3.65 | 4.52 | 4.35 | 5.79 |

**Table IIb** Specific localisation index of E48 $F(ab')_2$ in HNX-HN bearing mice

| Day 1 | Day 2 | Day 3 | Day 6 |
|---|---|---|---|
| 9.03 | 6.66 | 18.45 | 28.4 |

**Table IIIa** Tumour to tissue ratio of E48 and JSB-1 IgG in VX-A431 bearing mice

| | Tumour to tissue ratio of E48 IgG | | | |
|---|---|---|---|---|
| | Day 1 | Day 2 | Day 3 | Day 4 |
| Blood | 0.3±0.1 | 0.3±0.1 | 0.6±0.1 | 1.5±0.6 |
| Sternum | 3.3±0.9 | 3.6±0.8 | 5.9±0.8 | 12.2±4.0 |
| Liver | 1.3±0.3 | 1.5±0.5 | 2.2±0.4 | 6.6±2.5 |
| Spleen | 1.8±0.4 | 1.9±0.3 | 3.0±0.3 | 10.0±4.6 |
| Kidney | 1.3±0.1 | 1.4±0.3 | 2.1±0.5 | 6.5±4.1 |
| Colon | 4.0±0.4 | 4.2±1.3 | 6.3±1.1 | 15.6±7.0 |
| | Tumour to tissue ratio of JSB-1 IgG | | | |
| | Day 1 | Day 2 | Day 3 | Day 4 |
| Blood | 0.4±0.3 | 0.3±0.1 | 0.4±0.1 | 0.7±0.2 |
| Sternum | 4.6±3.9 | 3.7±0.7 | 4.9±1.0 | 6.5±1.1 |
| Liver | 1.7±1.1 | 1.5±0.2 | 1.8±0.5 | 3.7±0.9 |
| Spleen | 2.5±2.2 | 1.9±0.3 | 2.3±0.3 | 5.5±1.9 |
| Kidney | 1.9±1.5 | 1.3±0.1 | 1.7±0.5 | 3.5±1.2 |
| Colon | 5.2±3.8 | 4.9±1.1 | 5.3±1.2 | 9.0±2.8 |

**Table IIIb** Tumour to tissue ratio of E48 and JSB-1 $F(ab')_2$ in VX-A431 bearing mice

| | Tumour to tissue ratio of E48 $F(ab')_2$ | | | |
|---|---|---|---|---|
| | Day 1 | Day 2 | Day 3 | Day 4 |
| Blood | 3.2±0.4 | 5.7±2.0 | 7.3±0.6 | 3.5±3.0 |
| Sternum | 28.9±3.7 | 51.1±16.5 | 72.7±15.2 | 39.1±23.8 |
| Liver | 8.8±1.7 | 16.8±6.1 | 24.7±2.2 | 12.9±9.9 |
| Spleen | 12.7±1.6 | 23.5±5.2 | 38.9±5.8 | 17.2±8.7 |
| Kidney | 6.4±1.6 | 13.5±5.0 | 20.4±2.4 | 11.6±6.9 |
| Colon | 25.7±4.9 | 43.4±24.2 | 75.2±11.6 | 42.6±20.3 |
| | Tumour to tissue ratio of JSB-1 $F(ab')_2$ | | | |
| | Day 1 | Day 2 | Day 3 | Day 4 |
| Blood | 0.7±0.1 | 1.1±0.2 | 1.5±0.4 | 0.6±0.4 |
| Sternum | 8.7±1.6 | 12.3±2.4 | 17.1±5.3 | 6.5±3.6 |
| Liver | 2.5±0.2 | 4.2±0.9 | 6.2±1.3 | 2.8±1.8 |
| Spleen | 3.4±0.3 | 5.9±1.2 | 8.8±2.7 | 3.5±1.4 |
| Kidney | 1.5±0.4 | 2.6±0.5 | 4.0±0.9 | 2.1±0.9 |
| Colon | 7.8±2.2 | 10.8±5.5 | 19.7±6.9 | 6.7±3.5 |

**Table IVa** Specific localisation index of E48 IgG in VX-A431 bearing mice

| Day 1 | Day 2 | Day 3 | Day 6 |
|---|---|---|---|
| 1.71 | 1.14 | 1.20 | 1.03 |

**Table IVb** Specific localisation index of E48 $F(ab')_2$ in VX-A431 bearing mice

| Day 1 | Day 2 | Day 3 | Day 6 |
|---|---|---|---|
| 4.10 | 3.48 | 2.84 | 2.30 |

(m.t.w. ± s.e.m.): 352.6 ± 207.5), 11.9 (m.t.w. ± s.e.m.: 487.3 ± 238.0), 10.2 (m.t.w. ± s.e.m.: 398.6 ± 181.8) and 14.6 (m.t.w. ± s.e.m.: 326.8 ± 268.4) respectively (Figure 3a), whereas percentage ID/g in tumours of $^{131}$I E48 $F(ab')_2$ injected mice decreased after day 1 from 7.2 (m.t.w. ± s.e.m.: 277.4 ± 185.3) to 4.4 (m.t.w. ± s.e.m.: 368.5 ± 256.2) at day 2, 3.7 (m.t.w. ± s.e.m.: 510.8 ± 316.5) at day 3 and 2.0 (m.t.w. ± s.e.m.: 336.7 ± 203.0) at day 6 (Figure 4a). To exclude the possibility that uptake might be due to non-specific protein trapping, an isoptye matched antibody and its $F(ab')_2$ fragment were included as a control. The control IgG and control $F(ab')_2$ did not show any specific accumulation, either in the tumours or in any organ (Figures 3b and 4b).

Table IIa and IIb show the specific localisation index (SLI) of E48 IgG and E48 $F(ab')_2$ fragments, calculated by dividing the percentage ID/g of specific IgG or $F(ab')_2$ fragment (E48) in the tumour by the percentage ID/g of control IgG or $F(ab')_2$ fragment (JSB-1). In the course of the experiment, SLI of E48 IgG did not change significantly (3.7 at day 1 to 5.8 at day 6), whereas SLI of E48 $F(ab')_2$ fragment reached a maximum of 28.4 at day 6.

*Tumour uptake of E48 IgG and E48 $F(ab')_2$ fragment in VX-A431 bearing mice* The percentage ID/g of $^{131}$I E48 IgG and $^{131}$I E48 $F(ab')_2$ fragment in xenografts and various organs was determined as above. Tables IIIa and IIIb show the tumour to tissue ratios of E48 IgG and E48 $F(ab')_2$ fragment. At day 1, 2, 3 and 7, the percentage ID/g in tumours of E48 IgG injected mice was 3.7 (m.t.w. ± s.e.m.: 370.3 ± 247.2), 3.3 (m.t.w. ± s.e.m.: 525.3 ± 237.5), 3.5 (m.t.w. ± s.e.m.: 409.3 ± 142.9) and 3.0 (m.t.w. ± s.e.m.: 356.3 ± 248.1), respectively (data not shown), whereas percentage ID/g in E48 $F(ab')_2$ injected mice decreased steadily after day 1, from 2.4 (m.t.w. ± s.e.m.: 640.4 ± 573.6) to 1.4 (m.t.w. ± s.e.m.: 621.2 ± 605.6), 0.9 (m.t.w. ± s.e.m.: 274.6 ± 145.9) and 0.2 (m.t.w. ± s.e.m.: 290.8 ± 135.0), respectively (Figure 5a). Table IVa and IVb show the SLI of E48 IgG and E48 $F(ab')_2$ fragment. E48 IgG did not show a pronounced specific localisation in the tumour xenografts, as reflected by the low SLI of E48 IgG (Table IVa). E48 $F(ab')_2$

**Figure 5** Biodistribution data for **a** 10 $\mu$Ci $^{131}$I-labelled E48 F(ab')$_2$ and **b** 10 $\mu$Ci $^{125}$I-labelled JSB-1 F(ab')$_2$ in athymic mice bearing VX-A431 xenografts. At 1 (black), 2 (hatched), 3 (dotted) and 7 (open) days following i.v. injection tissues were dissected and counted and the percentage injected dose per gram (percentage ID/g) was calculated. Each day 3–4 mice were dissected.

however did show appreciable specific localisation (Table IVb), with SLI being 4.1 at its maximum at day 1. The control IgG (data not shown) and control F(ab')$_2$ (Figure 5b) did not show any specific accumulation in the tumours nor in any organ.

*Uptake in other organs* The percentage of E48 IgG and E48 F(ab')$_2$ fragment in various organs of HNX-HN bearing mice is presented in Figure 3a and Figure 4a. Only the most relevant organs are shown, the uptake in heart, stomach, jejenum, muscle, lung and tongue being comparable to or lower than uptake in colon. Sternum is shown because of the presence of haemopoietic tissue. The percentage ID/g of IgG (data not shown) and E48 F(ab')$_2$ (Figure 5a) in various organs of VX-A431 bearing mice was essentially the same as for HNX-HN bearing mice.

Control IgG or control F(ab')$_2$ fragment did not show any preferential localisation (Tables Ia, Ib, IIa and IIb and Figures 3b, 4b and 5b).

### *Radioimmunoscintigraphy*

*HNX-HN mice* E48 IgG and E48 F(ab')$_2$ injected mice were scanned at several time points following i.v. injection. After scanning, mice were dissected and biodistribution data were collected and compared to immunoscintigraphic images. Each picture in Figures 6a–d, Figures 7a and 7b and Figures 8a and 8b thus represent a different pair of mice being scanned. Orientation of the left mouse in each picture was head to tail from top to bottom, orientation of the right mouse was tail to head from top to bottom. Figure 6a represents two mice, scanned 1 day after injection of 10 $\mu$Ci $^{131}$I-labelled E48 IgG per mouse. The percentage ID/g of the xenografts was 10.13, 8.69, 11.87 and 10.49 from left to right respectively. High blood pool activity, most prominent in the thoracic cavity, hampered distinction of xenografts on day 1 (Figure 6a) as well as day 2 (Figure 6b). This background activity only markedly decreased 2 days after injection, after which timepoint xenografts could be visualised without appreciable background disturbance (Figure 6c, Figure 6d).

**Figure 6** Whole body scintigraphic images of pairs of athymic mice each bearing one or two subcutaneous HNX-HN xenografts given an injection of 10 $\mu$Ci $^{131}$I E48 IgG. Images were taken at day 1 **a**, day 2 **b**, day 3 **c** and day 6 **d**. Weight of xenografts in mg from left to right: **a**: 577, 575, 463, 464; **b**: 772, 211, 148, 341; **c**: 297, 472, 255, 694; **d**: 252, 379, NX, 766 (NX = no xenograft present).

Figures 7a and b represent mice being scanned 1 day and 2 days after injection of 10 $\mu$Ci $^{131}$I-labelled E48 F(ab')$_2$ fragment per mouse. Percentage ID/g at day 1 was of 6.09, 6.12, 8.28 and 9.03 from left to right, respectively (Figure 7a), and at day 2 percentage ID/g was 4.28, 6.11 and 3.24 and 2.89 from left to right, respectively (Figure 7b). No background activity could be detected, resulting in images showing only xenograft localisation of radioisotope.

*VX-A431 mice* Images of mice injected with 10 $\mu$Ci $^{131}$I-labelled E48 IgG did not result in identification of xenografts at any timepoint after injection, due to consistent high blood pool activity (images not shown). Images of mice injected

**Figure 7** Whole body scintigraphic images of pairs of athymic mice each bearing one or two subcutaneous HNX-HN xenografts given an injection of 10 $\mu$Ci $^{131}$I E48 F(ab′)$_2$. Images were taken at day 1 **a**, day 2 **b**. Weight of xenografts in mg from left to right: **a**: 314, 294, 205, 705; **b**: 895, 353, 585, 167.

**Figure 8** Whole body scintigraphic images of pairs of athymic mice each bearing one or two subcutaneous VX-A431 xenografts given an injection of 10 $\mu$Ci $^{131}$I E48 F(ab′)$_2$. Images were taken at day 1 **a**, day 2 **b**. Weight of xenografts in mg from left to right: **a**: 283, 1399, 442, 1810; **b**: 862, 698, 1677, NX.

with 10 $\mu$Ci $^{131}$I-labelled E48 F(ab′)$_2$ however, 1 day (Figure 8a) and 2 days (Figure 8b) after injection, did show xenografts depicted without significant background disturbance. Percentage ID/g at day 1 was 2.41, 1.72, 2.16 and 2.08 from left to right, respectively (Figure 8a) and at day 2 percentage ID/g was 1.06, 1.11 and 1.03, respectively (Figure 8b).

### Discussion

Conjugates of radioisotopes and immunoglobulines or their fragments are attractive agents for tumour diagnosis and therapy. However, fragments are cleared more rapidly from the blood than whole IgG (Pervez *et al.*, 1988; Buchegger *et al.*, 1986; Endo *et al.*, 1988; Colapinto *et al.*, 1988), resulting in lower background activity and reducing the radiation dose to normal tissues. Moreover, fragments lack the Fc region responsible for nonspecific tissue uptake by Fc receptor binding and may be less immunogenic in humans (Smith *et al.*, 1979). Finally, the smaller size of fragments should allow better tumour penetration than whole IgG. Although these phenomena hold true for both Fab and F(ab′)$_2$, reports dealing with studies on tumour uptake of whole IgG, F(ab′)$_2$ and Fab often show a strong decrease in tumour uptake of F(ab′)$_2$ and Fab when compared to whole IgG and a strong decrease in tumour to normal tissue ratios of Fab when compared to F(ab′)$_2$, most likely the result of increased clearance from the blood and a decreased affinity inherent in the generation of univalent Fab fragments (Buchegger *et al.*, 1986; Colapinto *et al.*, 1988; Endo *et al.*, 1988; Wahl *et al.*, 1983).

In this investigation we compared the characteristics of E48 IgG and E48 F(ab′)$_2$ with regard to biodistribution and imaging parameters in nude mice bearing SCC xenografts. In a previous study we reported the production of MAb E48 recognising a 22 kDa antigen exclusively expressed in stratified squamous epithelia and transitional epithelium of the bladder (Quak *et al.*, 1990*a*,*b*). In neoplastic tissues, reactivity with MAb E48 is restricted to squamous cell carcinoma of head and neck, lung, cervix and skin and to urinary bladder carcinoma. Reactivity was observed mainly on the membrane and to a lesser extent within the cytoplasm. Based on immunohistochemical examination, two xenograft lines were selected from 8 SCC lines available at our laboratory. The head and neck xenograft line SCC HNX-HN revealed strong and homogenous membrane binding of the antibody and showed a well organised tumour structure with separated tumour nests in well developed stroma, representing a pattern displayed by the majority of human tumours investigated sofar. The vulva SCC xenograft line VX-A431 revealed a moderate and diffuse binding pattern of the antibody and displayed a much less organised tumour tissue structure. These two tumours represent the extremes of immunohistochemical reaction patterns as observed in 92 human tumours immunohistochemically stained with MAb E48. In a previous study, we already demonstrated the capacity of E48 IgG for specific delivery of radioisotopes to tumours in nude mice bearing a SCC xenograft line (Quak *et al.*, 1989). In nude mice bearing the HNX-HN xenograft line, the use of the F(ab′)$_2$ fragment of E48 strongly improved tumour uptake ratios and localisation specificity when compared directly with IgG. Although the digestion of IgG for the generation of F(ab′)$_2$ did not significantly alter the affinity of the radiolabelled conjugate, F(ab′)$_2$ did show a decrease in percentage ID/g tumour tissue as compared to IgG. Differences in pharmacokinetics of the conjugates are likely to be the major factors leading to this decrease. However, the improved tumour uptake ratios and specificity of localisation resulted in images without background disturbance at day 1 for F(ab′)$_2$, whereas IgG did not give comparable images until day 3. In nude mice bearing the xenograft line VX-A431, E48 IgG uptake in tumours did not differ from control IgG, thus failing to reach tumour to non tumour ratios enabling visualisation of tumour xenografts at any time point. E48 F(ab′)$_2$ fragments however did show specific localisation in tumour tissue, and although the percentage ID/g was almost one third of the percentage ID/g of E48 $F(ab')_2$ in HNX-HN bearing mice and the SLI of $F(ab')_2$ in VX-A431 bearing mice was less than half the SLI of $F(ab')_2$ in HNX-HN bearing mice, tumours could still well be visualised at day 1. These differences in percentage ID/g and specificity of localisation between the HNX-HN and VX-A431 xenograft lines might be due to such variables as vascularisation, blood vessel morphology and permeability, tumour microcirculation, necrosis, composition of extracellular matrix and intratumoural hydrostatic pressure, parameters likely to be of considerable influence on the efficacy of non-surgical modalities (Sands *et al.*, 1988; Cobb, 1989; Kallinowski *et al.*, 1989; Jain & Wie, 1977; Sweet *et al.*, 1979; Hori *et al.*, 1986).

Additionally, differences in number and exposition of antigenic sites cannot be ruled out as major factors in causing the differences in localisation of the radiolabelled conjugates between the two xenograft lines. A lower number of exposed antigenic sites per cell in the VX-A431 xenograft line as compared to the HNX-HN xenograft line combined with the higher penetration capacities of the small E48 $F(ab')_2$ fragment as compared to E48 IgG might very well explain the inability of E48 IgG to localise specifically in the VX-A431 xenograft line. In this perspective, the differences in localisation characteristics between the HNX-HN and the VX-A431 xenograft lines might reflect the heterogeneity of expression and accessibility of antigenic sites in human tumours in the clinical situation.

In normal tissues, neither E48 IgG nor E48 $F(ab')_2$ showed any non specfic accumulation in vital organs, including the radiation sensitive reticuloendothelial system (liver, spleen and bone marrow). The overall tumour to non-tumour ratios of $F(ab')_2$ however were several times higher than tumour to non tumour ratios of IgG. Tumour to non-tumour ratios of $F(ab')_2$ in VX-A431 bearing mice were still higher than tumour to non-tumour ratios of IgG in HNX-HN bearing mice.

So far, only a limited number of MAb's reacting with human squamous cell carcinoma have been described. Most of these antibodies show considerable cross reactivity with other tissues or only show reactivity with SCC of distinct sites of tissue origin. Additionally, features of these antibodies as isotype, cytoplasmatic localisation of the antigen and relatively low affinity of the antibody render them less suited for *in vivo* localisation studies or application in a clinical setting.

Within the limitations of our experiments, we have shown the E48 $F(ab')_2$ fragment to be a promising candidate for immunodiagnostic application in a clinical setting. Preparations for a phase I/II clinical study are currently in progress.

The authors wish to thank Dr van Lingen of the Department of Nuclear Medicine for help with image analysis.

This work was supported in part by Centocor Europe Inc., Leiden, The Netherlands.

### References

BOEHEIM, K., SPEAK, J.A., FREI, E. & BERNAL, S.D. (1985). SQM1 antibody defines a surface membrane antigen in squamous carcinoma of the head and neck. *Int. J. Cancer*, **36**, 137.

BADGER, C.C., KROHN, K.A. & BERNSTEIN, I.D. (1987). *In vitro* measurement of avidity of radioiodinated antibodies. *Int. J. Radiat. Appl. Instrum.*, **14**, 605.

BRENNER, B.G., JOTHY, S., SHUSTER, J. & FUKS, A. (1982). Monoclonal antibodies to human long-tumor antigens demonstrated by immunofluorescence and immunoprecipitation. *Cancer Res.*, **42**, 3187.

BUCHEGGER, F., MACH, J.-P., LEONNARD, P. & CARREL, S. (1986). Selective tumor localisation of radiolabelled anti-human melanoma monoclonal antibody fragment demonstrated in the nude mouse model. *Cancer*, **58**, 655.

CAREY, T.E., KIMMEL, K.A., SCHWARTZ, D.R., RICHTER, D.E., BAKER, S.R. & KRAUSE, C.J. (1983). Antibodies to human squamous cell carcinoma. *Otolaryngol. Head & Neck Surg.*, **91**, 482.

COBB, L.M. (1989). Intratumour factors influencing the access of antibody to tumour cells. *Cancer Immunol. Immunother.*, **28**, 235.

COLAPINTO, E.V., HUMPHREY, P.A., ZALUTSKY, M.R. & 5 others (1988). Comparative localization of murine monoclonal antibody MEL-14 $F(ab')_2$ fragment and whole IgG2a in human glioma xenografts. *Cancer Res.*, **48**, 5701.

ENDO, K., KAMMA, H. & OGATA, T. (1988). Radiolabelled monoclonal antibody 15 and its fragments for localization and imaging of xenografts of human lung cancer. *J. Natl Cancer Inst.*, **80**, 835.

FERNSTEN, P.D., PEKNY, K.W., REISFELD, R.A. & WALKER, L.E. (1986). Antigens associated with human squamous cell lung carcinoma defined by murine monoclonal antibodies. *Cancer*, **46**, 2970.

HAISMA, H.J., HILGERS, J. & ZURAWSKI, V.R. (1986). Iodination of monoclonal antibodies for diagnosis and therapy using a convenient one vial method. *J. Nucl. Med.*, **27**, 1890.

HORI, K., SUZUKI, M. & SAITO, A.I. (1986). Increased tumour pressure in association with the growth rate of rat tumours. *Jpn. J. Cancer Res.*, **77**, 65.

JAIN, R.K. & WIE, J. (1977). Dynamics of drug transport in solid tumours: distrubuted parameter model. *J. Bioeng.*, **1**, 3.

KALLINOWSKI, F., SCHLENGER, K.H., RUNKEL, S. & 4 others (1989). Blood flow, metabolism, cellular environment, and growth rate of human tumour xenografts. *Cancer Res.*, **49**, 3759.

KIMMEL, K.A. & CAREY, T.E. (1986). Altered expression in squamous carcinoma cells of an orientation-restricted epithelial antigen detected by monoclonal antibody A9. *Cancer Res.*, **46**, 3614.

KOPROWSKA, I., ZIPFEL, S., ROSS, A.H. & HERLYN, M. (1986). Development of monoclonal antibodies that recognize antigens associated with human cervical carcinoma. *Acta Cytol.*, **30**, 207.

KYOIZUMI, S., AKIYAMA, M., KOUNO, N. & 4 others (1985). Monoclonal antibodies to human squamous-cell carcinoma of the lung and their application to tumor diagnosis. *Cancer Res.*, **45**, 3274.

LINDMO, T., BOVEN, E. & LUTTITA, F. (1984). Determination of the immunoreactive fraction of radiolabelled monoclonal antibodies by linear extrapolation to binding at infinite antigen excess. *J. Imm. Methods*, **72**, 77.

MYOKEN, Y., MOROYAMA, T., MIYAUCHI, S., TAKADA, K. & NAMBA, M. (1987). Monoclonal antibodies against oral squamous-cell carcinoma reacting with keratin proteins. *Cancer*, **60**, 2927.

PERVEZ, S., EPENETOS, A.A., MOOI, W.J. & 4 others (1988). Localization of monoclonal antibody AUA1 and its $F(ab')_2$ fragment in human tumor xenografts: an autoradiographic and immunohistochemical study. *Int. J. Cancer*, Suppl. 3, 23.

QUAK, J.J., BALM, A.J.M., BRAKKEE, J.G.P. & 5 others (1989). Localization and imaging of radiolabelled monoclonal antibody against squamous-cell carcinoma of the head and neck in tumor bearing nude mice. *Int. J. Cancer*, **44**, 534.

QUAK, J.J., BALM, A.J.M., VAN DONGEN, G.A.M.S. & 4 others (1990*a*). A 22-kD surface antigen detected by monoclonal antibody E48 is exclusively expressed in stratified squamous and transitional epithelia. *Am. J. Path.*, **136**, 191.

QUAK, J.J., GERRETSEN, M., SCHRIJVERS, A., MEIJER, C.J.L.M., VAN DONGEN, G.A.M.S. & SNOW, G.B. (1990*b*). Detection of squamous cell carcinoma xenografts in nude mice by radiolabeled monoclonal antibody E48. *Arch Otolaryngol. Head & Neck Surg.* (in the press).

RANKEN, R., WHITE, C.F., GOTTFRIED, T.G. & 5 others (1987). Reactivity of monoclonal antibody 17.13 with human squamous cell carcinoma and its application to tumour diagnosis. *Cancer Res.*, **47**, 5684.

SAMUEL, J., NOUJAIM, A.A., WILLANS, D.J., BRZEZINSKA, G.S., HAINES, D.H. & LONGENECKER, B.M. (1989). A novel marker for basal (stem) cells of mammalian stratified squamous epithelia and squamous cell carcinomas. *Cancer Res.*, **49**, 2465.

SANDS, H., JONES, P.L., SHAH, S.A., PALME, D., VESSELLA, R.L. & GALLAGHER, B.M. (1988). Correlation of vascular permeability and blood flow with monoclonal antibody uptake by human Clouser and renal cell xenografts. *Cancer Res.*, **48**, 188.

SCHEPER, R.J., BULTE, J.W.M., BRAKKEE, J.G.P. & 8 others (1988). Monoclonal antibody JSB-1 detects a highly conserved epitope on the P glycoprotein associated with multidrug resistance. *Int. J. Cancer*, **42**, 389.

SMITH, T.W., LLOYD, B.L., SPICER, N. & HABER, E. (1979). Immunogenicity and kinetics of distribution and elimination of sheep digoxin-specific IgG and Fab fragments in the rat and baboon. *Clin. Exp. Immunol.*, **36**, 384.

SWEET, M.B.E., THONAR, E.J.M., BERSON, S.D., SKIKNE, M.I., IMMELMAN, A.R. & KERR, W.A. (1979). Biochemical studies on the matrix of cranivertebral chordoma and a metastasis. *Cancer*, **44**, 652.

TATAKE, R.J., AMIN, K.M., MANIAR, H.S., JAMBHEKAR, N.A., SRIKHANDE, S.S. & GANGAL, S.G. (1989). Monoclonal antibody against human squamous-cell-carcinoma-associated antigen. *Int. J. Cancer*, **44**, 840.

WAHL, R.L., PARKER, C.W. & PHILLPOTT, G.W. (1983). Improved radioimaging and tumor localization with monoclonal $F(ab')_2$. *J. Nucl. Med.*, **24**, 316.

ZENNER, H.P. & HERMANN, I.W. (1981). Monoclonal antibodies against surface antigens of laryngeal carcinoma cells. *Arch. Otolaryngol.*, **233**, 161.